# Chitosan–Type-A-Gelatin Hydrogels Used as Potential Platforms in Tissue Engineering for Drug Delivery

**DOI:** 10.3390/gels10070419

**Published:** 2024-06-26

**Authors:** Hanaa Mehdi-Sefiani, Carmen Mª Granados-Carrera, Alberto Romero, Ernesto Chicardi, Juan Domínguez-Robles, Víctor Manuel Perez-Puyana

**Affiliations:** 1 Department of Engineering and Materials Science and Transportation, University of Seville, 41012 Seville, Spain; 2 Department of Chemical Engineering, Faculty of Chemistry, University of Seville, 41012 Seville, Spain; 3 Department of Pharmacy and Pharmaceutical Technology, Faculty of Pharmacy, University of Seville, 41012 Seville, Spain

**Keywords:** chitosan, type A gelatin, hydrogels, characterization, drug-delivery

## Abstract

Hydrogels are materials made of crosslinked 3D networks of hydrophilic polymer chains that can absorb and retain significant amounts of water due to their hydrophilic structure without being dissolved. In relation to alternative biomaterials, hydrogels offer increased biocompatibility and biodegradability, giving them distinct advantages. Thus, hydrogel platforms are considered to have the potential for the development of biomedical applications. In this study, the main objective was the development of hybrid hydrogels to act as a drug delivery platform. These hydrogels were made from chitosan (CH) and type A gelatin (G), two natural polymers that provide a supportive environment for cellular attachment, viability, and growth, thanks to their unique properties. Particularly, the use of gelatins for drug delivery systems provides biodegradability, biocompatibility, and non-toxicity, which are excellent properties to be used in the human body. However, gelatins have some limitations, such as thermal instability and poor mechanical properties. In order to improve those properties, the aim of this work was the development and characterization of hybrid hydrogels with different ratios of CH–G (100–0, 75–25, 50–50, 25–75, 0–100). Hydrogels were characterized through multiple techniques, including Fourier transform infrared (FTIR) spectroscopy, rheological and microstructural studies, among others. Moreover, a model hydrophilic drug molecule (tetracycline) was incorporated to evaluate the feasibility of this platform to sustain the release of hydrophilic drugs, by being tested in a solution of Phosphate Buffer Solution at a pH of 7.2 and at 37 °C. The results revealed that the synergy between chitosan and type A gelatin improved the mechanical properties as well as the thermal stability of it, revealing that the best ratios of the biopolymers are 50–50 CH–G and 75–25 CH–G. Thereby, these systems were evaluated in a controlled release of tetracycline, showing a controlled drug delivery of 6 h and highlighting their promising application as a platform for controlled drug release.

## 1. Introduction

A drug delivery system (DDS) is defined as the technology that introduces an adequate dosage of therapeutic substance into the body to achieve effectively and safely its appropriate therapeutic effect [1]. Thus, the main objective of a DDS is to ensure that drugs are administered in the required place in the body and at the correct time, avoiding adverse effects [2]. However, conventional DDSs (tablets, capsules, syrups, etc.) have limitations that are mainly attributable to their poor bioavailability, fluctuations of the drug concentration in plasma, limited solubility, poor tissue distribution of drug particles, and uncontrollable drug release characteristics [1,3,4]. Consequently, to establish new methods for achieving controlled and targeted drug release, the design and development of new DDSs have been considered [5]. In fact, chemists and chemical engineers are contributing to improve the development of controlled drug delivery technologies, which have numerous advantages such as efficacy, less toxicity, and the improvement of patient compliance and convenience [6].

A DDS includes a wide variety of methods, including liposomes, micelles, and nanoparticles [2]. One way to achieve a controlled DDS is the development of an adequate encapsulation system that provides protection for the drug, preventing the surrounding environment of the body from carrying its uncontrolled release or out-of-place activation [7]. Over the last few years, biomaterials have attracted interest in the development of controlled DDSs. Particularly, hydrogels have been demonstrated as the ideal pharmaceutical carrier in biomedical areas due to their unique physical properties, such as porosity, biocompatibility, biodegradability, and nontoxicity, along with their controlled release properties and ability to protect drugs [8,9,10,11].

Furthermore, hydrogels are one of the outstanding classes of polymer-based drug-controlled release systems for their unique pharmacokinetic properties among various DDSs [12]. Thus, hydrogels have been widely useful due to their ability to manufacture and combine with natural and synthetic components, as well as the properties mentioned above [13]. Polymer-based hydrogels are three-dimensional (3D) networks of hydrophilic polymer that possess the capacity to maintain their structural integrity while absorbing and retaining large amounts of water and biological fluids [8,14]. Moreover, as the DDS’s release mechanism would be affected by the properties of the hydrogel, such as porosity or swelling degree, polymer-based hydrogels have the capability to release the drugs gradually following a specific release kinetic achieved by modulating their compositions and structures [14,15] Furthermore, it can be highlighted that currently, the existence of “smart hydrogels” permit their use as sensors, thanks to their responsiveness towards stimuli such as temperature, solvents, pH, light, molecules, and mechanical force [13].

Recently, attention has been focused on finding materials that are non-toxic and biocompatible [16]. Particularly, natural polymers are materials obtained from human beings, plants, or animals, which have poor mechanical characteristics; but, due to their high affinity with water, gelling capacity, mucoadhesiveness, nontoxicity, and negative antigenicity in live organisms, they are widely used in tissue engineering (in biomedical applications, among others) [17,18]. Some examples of natural polymers, such as collagen, gelatin, alginate, and chitosan, can be mentioned [19]. Numerous strategies have been used to develop natural polymeric-based hydrogels with improved mechanical properties; among them, we can distinguish double networking [20], physical, enzymatic, or chemical crosslinking [18], and electrochemistry functionalization [21].

On the one hand, chitosan (CH) is a natural polymer derived from the deacetylation of chitin, which is present in the exoskeletons of crustaceans. Chitosan and its derivatives have become promising biological materials due to their distinctive molecular structure, excellent biological activities, and their properties such as biocompatibility, biodegradability, cytocompatibility, non-allergenicity, non-toxicity, and mucoadhesiveness, as well as their antitumor, antioxidant, antibacterial, and antifungal activities [22,23,24,25,26,27]. The reactive functional groups found in chitosan play an important role in aiding the synthesis of three-dimensional hydrogel. Chitosan-based hydrogels have been widely used in medical and pharmaceutical fields for years. One of their advantages is their complex network architecture, which offers a versatile platform that allows the incorporation of various bioactive compounds [23]. Additionally, chitosan has the capability to improve the bioavailability and stability of multiple small molecule drugs, peptides, and proteins, as well as sustain drug release due to its mucoadhesive properties. Moreover, chitosan-based drug delivery systems can aim at specific cells or tissues by adding to them specific ligands or antibodies [24,27]. Furthermore, the properties of chitosan-based hydrogels can be further tuned by altering their composition or combining them with other natural or synthetic polymers [24]. For example, Vasile et al. [28] developed a chitosan composite incorporating ZnO nanoparticles. In this way, potential material for wound care was obtained that combines the antibacterial properties of their components with a controlled release of the antibiotic (gentamicin). In other research completed by Xu et al. [29], chemical chitosan and catechol hydrogels were crosslinked with genipin in order to study the release of lidocaine. The results showed that catechol increased the hydrogel density, and the drug was more effective. Moreover, Ren et al. [12] developed crosslinked injectable hydrogels by oxidizing the blend of chitosan, gelatin, and dopamine as the drug of the system. The results showed stable mechanical strength and good degradability.

Gelatin is a natural polymer obtained through hydrolytic degradation of type I collagen. This biopolymer has been widely used in biomedical applications due to its high biodegradability, biocompatibility, non-toxicity, non-immunogenicity, and highwater absorption [18,30,31,32]. Notably, these properties render gelatin an excellent candidate for drug-delivery vehicles [31]. Gelatin has recently become a significant factor in tailoring drug release in pharmaceutical formulations [18]. In fact, gelatin is the main ingredient in hard and soft capsules in pharmaceutical applications since it melts above 30 °C and easily releases drugs. Additionally, the hydrophilic nature of gelatin facilitates the permeation of body fluids into the particles, which leads to an increase in the diffusion-mediated release of drugs [30]. There are two types of gelatins, type A and type B gelatin, which are obtained by an acid-treated and alkali-treated process [31]. In particular, type A gelatin (G) has demonstrated great biocompatibility and the ability to form hydrogels [32]. According to the literature, gelatin hydrogels are a promising choice for delivering therapeutic or pharmaceutical compounds, thanks to their distinct properties. They can be designed to react to changes in temperature or pH levels, ensuring accurate drug release in response to specific bodily conditions. Furthermore, they are highly effective in delivering drugs to specific locations, which enhances their efficacy while minimizing side effects [18].

In particular, several investigations have exhibited the potential of chitosan and gelatin carriers in drug delivery systems [25]. First, Mahdizadeh et al. [33] developed a chitosan–gelatin carrier, which proved through drug release, release kinetics, and physical and mechanical studies its capability to be used as an insulin nanoparticle carrier. In another study, Mathew et al. [34] developed dopamine-loaded chitosan/gelatin nanocomposites that demonstrated to be excellent drug delivery carriers with optimal antioxidant and antibacterial properties because of their sustained release in a neutral medium, as well as their capability to inhibit the DPPH radical and Staphylococcus aureus. Finally, Wu et al. synthesized microspheres combining gelatin with an anti-cancer medication (pacific yew tree bark) and chitosan in order to achieve a high drug entrapment efficiency [26].

In this context, the purpose of this work was the development and characterization of chitosan–type-A-gelatin hydrogels by studying different biopolymeric ratios, as well as evaluating their rheological and microstructural properties. Moreover, the incorporation of a model hydrophilic drug molecule (tetracycline) to evaluate the feasibility of this platform to sustain the release of hydrophilic drugs. This research sets a strong foundation for further exploration into the efficacy and practicality of this novel approach to drug delivery and its potential application in tissue engineering.

## 2. Results and Discussion

### 2.1. Chemical Characterization: Fourier Transform Infrared Spectroscopy (FTIR)

**Figure 1:**
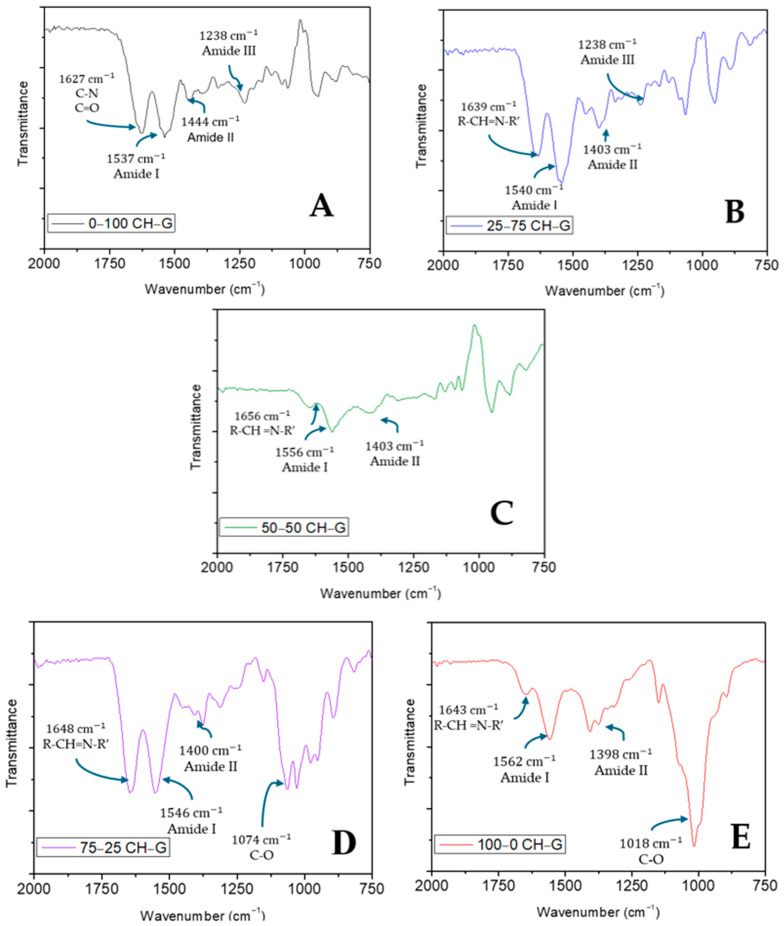
Fourier Transform Infrared Spectroscopy (FTIR) of the different hydrogels synthesized: (**A**) 0–100 CH–G; (**B**) 25–75 CH–G; (**C**) 50–50 CH–G; (**D**) 75–25 CH–G; (**E**) 100–0 CH–G.

FTIR characterization is necessary to ensure that the interactions between chitosan and type A gelatin occurred. Particularly, the peaks of the FTIR informed about the stretching and bending of the bounds. For this purpose, FTIR measurements of the different hydrogels were carried out and the results are shown in Figure 1.

Figure 1A shows the spectrum of the type A gelatin-based hydrogel. First, the presence of C-N and C=O bands occur in a frequency range between 1750 and 1600 cm^−1^. On the other hand, in a frequency range between 1600 and 1500 cm^−1^, a signal called Amide I is indicated, which is associated with the stretching of C=O. Then, Amide II signal appears in the range 1500–1375 cm^−1^ for N-H bending. N-H bending is also present in the range 1250–1125 cm^−1^ for the Amide III signal [35]. On the other hand, Figure 1E shows the profile of the chitosan-based hydrogel. In this case, in a frequency range between 1750 and 1600 cm^−1^, the presence of a CH=N band is indicated. Subsequently, the signal for C=O stretch (Amide I) occurs in the range 1600–1500 cm^−1^ and the signal for the N-H bending (Amide II) occurs in the range of 1500 and 1375 cm^−1^. Finally, in a range of 1125–1000 cm^−1^ the presence of a C-O band is indicated [36,37,38,39].

Figure 1B–D show the profiles of the binary systems. In the case of 25–75 CH–G (Figure 1B), it can be highlighted that the signal for C=O stretch (Amide I) occurs in the range 1600–1500 cm^−1^ and the signal for the N-H bending occurs in the range of 1500 and 1375 cm^−1^ (Amide II) and in the range of 1250–1125 cm^−1^ (Amide III) as it occurred in Figure 1A associated with the spectrum of the type A gelatin hydrogel. What is different is that the CH=N band appears in the range of 1750 and 1600 cm^−1^, which is characteristic of the chitosan-based hydrogel’s spectrum. On the other hand, the 75–25 CH–G spectrum (Figure 1D) indicates the presence of a CH=N band in a frequency range between 1750 and 1600 cm^−1^. Furthermore, the stretching of C=O (Amide I) in the range 1600–1500 cm^−1^ and N-H bending (Amide II) in the range 1500–1375 cm^−1^ are also indicated. Finally, a band of C-O occurs in the range 1125–1000 cm^−1^. In the 50–50 CH–G spectrum (Figure 1C), the spectrum of both biopolymers shows synergy, resulting in previously significant peaks being attenuated. The peak at 1012 cm^−1^ is similar to that of the type A based-hydrogel spectrum. The presence of a CH=N band in a frequency range between 1750 and 1600 cm^−1^ is also observed. Finally, the stretching of C=O (Amide I) in the range of 1600–1500 cm^−1^ and N-H bending (Amide II) in the range of 1500–1375 cm^−1^ can be seen [36,37,38].

### 2.2. Rheological Characterization of Hydrogels

Apart from evaluating the physicochemical properties of the hydrogels, rheological characterization was also carried out.

Firstly, strain sweep tests were performed in order to determine the linear viscoelastic interval, which is the interval where elastic and viscous moduli are independent of deformation as well as the critical deformation, which refers to the maximum deformation supported by the sample within the linear viscoelastic interval (Table 1) [40]. Attending to the critical deformation shown in Table 1, it can be noted that type A gelatin-based hydrogel has critical deformation values higher than the critical deformation values of chitosan-based hydrogel. This difference may be due to the fact that chitosan-based hydrogel is a rigid material that offers a specific structure, while the type A gelatin system is more deformable and allows its chains to be deformed without breaking them [41]. However, when the hydrogel has a higher proportion of chitosan (75–25 system), the system becomes rigid but improves the values obtained by the unitary chitosan system. Conversely, the systems with a higher proportion of type A gelatin show a deformable characteristic, which is better than values obtained by the unitary type A gelatin system studied at 5 °C. On the other hand, it can be highlighted that the temperature of measurements could affect the deformable capabilities of the unitary systems. In fact, the critical strain obtained at 5 °C does not follow a defined trend. Particularly, the unitary type A gelatin system evaluated at 5 °C shows a significant decrease in its deformable capability. In contrast, when the unitary chitosan system and the systems containing chitosan were assessed at low temperatures, they showed a slight increase in their deformable capability. Moreover, the critical strain of binary hydrogels increased in comparison to unitary hydrogels (100–0 CH–G and 0–100 CH–G) due to the synergistic effect produced by the combination of both biopolymers. So, it can be verified that the presence of gelatin in the structure increases the critical strain of the system. On the other hand, the critical strain obtained at 40 °C follows a defined trend. However, it can be noted that the increase in the proportion of type A gelatin induces a higher value of critical deformation. Finally, these values can be compared with those obtained in another work by Sánchez-Cid et al. [41]. Specifically, the critical deformation values of unitary chitosan-based hydrogel obtained at 5 °C are too similar to those obtained in this work.

**Figure 2:**
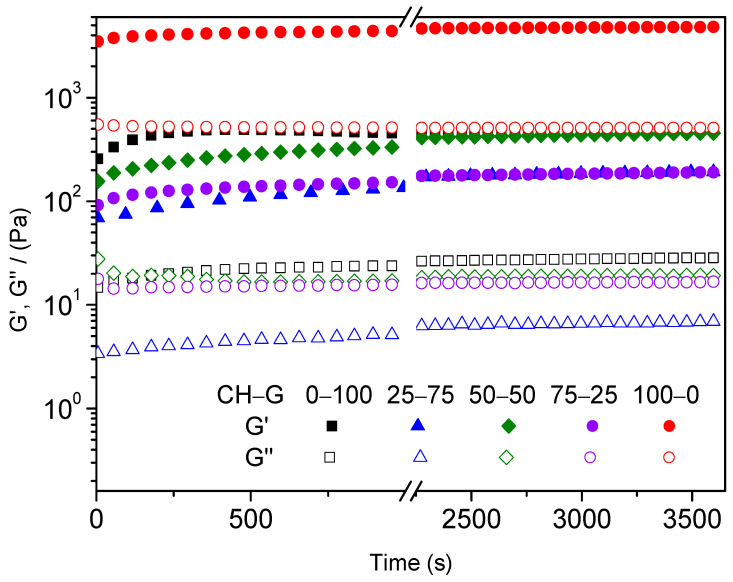
Time sweep test of chitosan–type-A-gelatin hydrogels during the gelation process.

After the critical deformation of each system was obtained, a time sweep test was carried out before gelation to analyze the evolution of both elastic (G′) and viscous (G″) moduli during the gelation process. Figure 2 shows how the gelation process increases the elastic modulus (G′) of the different hydrogels evaluated. Moreover, the distance between G′ and G″ increases with time, which indicates that the systems are gaining a more solid character. However, after one hour, the values of the elastic modulus (G′) and the viscous modulus (G″) became constant.

**Figure 3:**
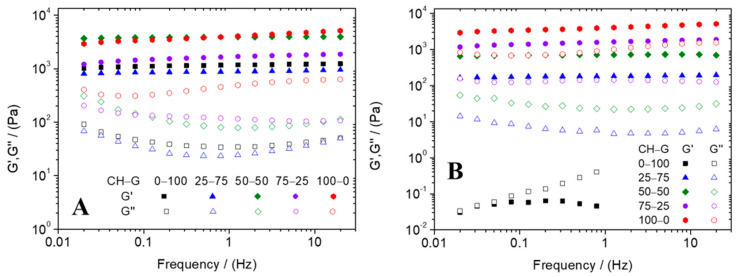
Frequency sweep test of chitosan–type-A-gelatin hydrogels evaluated at 5 °C (**A**) and 40 °C (**B**).

Subsequently, frequency sweep tests were also carried out at different temperatures, and the results are shown in Figure 3. Firstly, it is important to note that Figure 3A,B present the profile of elastic (G′) and viscous (G″) moduli in the frequency range studied of the different systems at 5 °C and 40 °C, respectively. According to the profiles obtained for the five systems, G′ values are always higher than G‘’ values, thus showing a predominantly solid behavior, which also indicates the elastic response of the hydrogel system. Furthermore, a constant profile in the frequency range corroborates the stability of the systems. On the other hand, related to the temperature of measurements, it can be noted that at high temperatures (Figure 3B), the presence of a higher proportion of gelatin leads to an increase in the values of G′ and G″. However, the evolution of the 0–100 CH–G system at 40 °C is not the same as the other ones, showing the low stability of type A gelatin at high frequencies. Thus, it is important to note that an increase in the proportion of chitosan leads to an increase in viscoelastic parameters due to its structural characteristics [42]. Comparing the elastic modulus and loss tangent at 1 Hz included in Table 1, it can be observed that the G′ values of the systems generally increased with a lower temperature of measurement and the presence of a high proportion of chitosan. Moreover, the tan (δ)_1_ values shown in Table 1 indicate that using a higher temperature of measurement, the systems showed a decrease in their solid character since the tan δ value increase suggests that the value of G″ is greater than the G′. Furthermore, the tan δ values decrease with the presence of chitosan, indicating that with a higher proportion of chitosan, the systems obtained had higher solid character. These differences highlight the better mechanical resistance of hydrogels that have a small amount of chitosan due to the necessity of a material that can withstand mechanical stress during the cell growth and proliferation stage. Finally, highlighted are the similarities between the profile of the frequency curve of the unitary type A gelatin-based hydrogel obtained at 5 °C and the one obtained in research done by Mehdi-Sefiani, H. et al. [32]. On the other hand, we found similarities between the frequency curves obtained for binary hydrogels made by chitosan and the collagen studied by Sánchez-Cid et al. [40].

**Figure 4:**
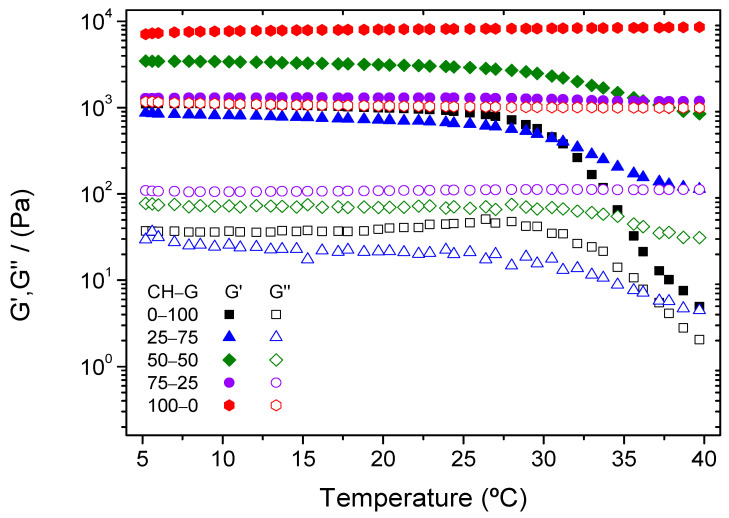
Temperature ramp test of chitosan–type-A-gelatin-based hydrogels.

Subsequently, temperature ramps were evaluated, and the results are shown in Figure 4. Firstly, it can be noted that the chitosan-based hydrogels are thermal stable compared with hydrogels that were developed only with type A gelatin. To corroborate this information, the critical temperature of each system was obtained and included in Table 1**.** The value of the critical temperature also indicated that hydrogels with a higher proportion of chitosan or unitary chitosan hydrogel have greater thermal stability than hydrogels elaborated with a higher proportion of type A gelatin. It can also be attributed to gelatin melting above 30 °C [30].

**Figure 5:**
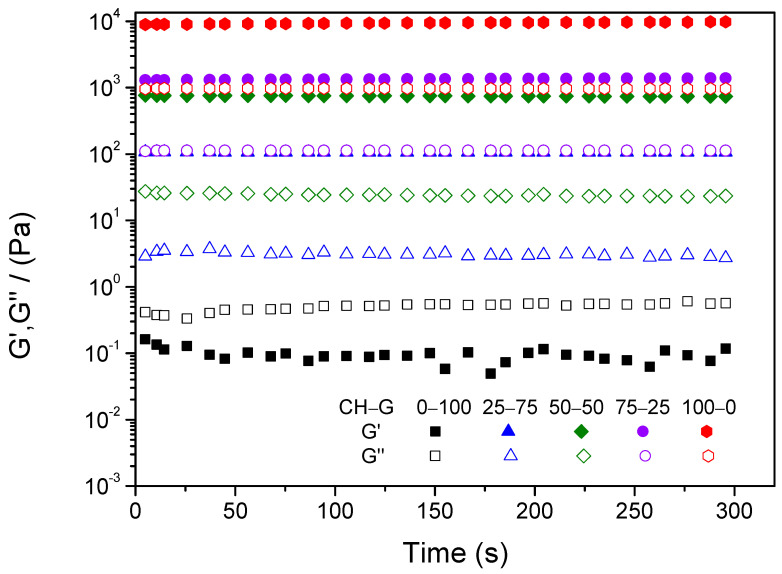
Time sweep test of chitosan–type-A-gelatin-based hydrogels at 40 °C.

Furthermore, with the aim of studying if these hydrogels are capable of being used in medical applications, time sweep tests were further evaluated by maintaining the temperature at 40 °C for a period of 1 h. As can be seen in Figure 5, all the systems remained stable at 40 °C except the type A gelatin unitary hydrogel, which showed instability because of its degradation at temperatures above 30 °C, as noted in Figure 4.

### 2.3. Microstructural Characterization: Scanning Electron Microscopy (SEM)

**Figure 6:**
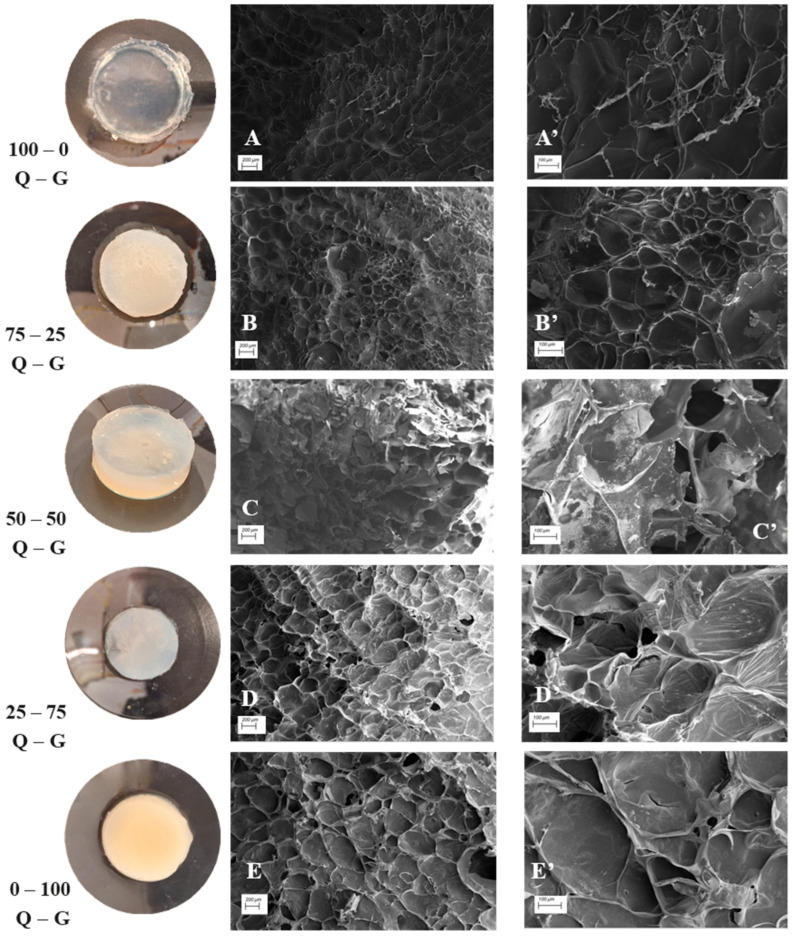
Superficial and cross-sectional SEM images of chitosan-based hydrogel ((**A**) and (**A′**), respectively), 75–25 CH–G hydrogels ((**B**) and (**B′**), respectively), 50–50 CH–G hydrogels ((**C**) and (**C′**), respectively), 25–75 CH–G hydrogels ((**D**) and (**D′**) respectively) and type A gelatin-based hydrogel ((**E**) and (**E′**), respectively). Macroscopic images of each hydrogel have also been included.

In order to study the microstructural characteristics of the different systems of hydrogels, a SEM imaging was obtained (Figure 6).

First, a big difference between the structure of the chitosan-based hydrogel (Figure 6A,A′) and the type A gelatin-based hydrogel (Figure 6E,E′) can be noted. The chitosan-based hydrogel has an ordered sponge network structure consisting of small spheres (aggregates) linked together, while the structure of the type A gelatin-based hydrogel is characterized by the presence of porous grains as shown in other studies [32,43,44]. However, comparing the porosity of both systems can highlight the lower porosity in the structure of chitosan-based hydrogels, which corroborates the dates obtained in the rheology about its greater structuring and, consequently, its solid characteristic. Binary systems present a combined structure that is really affected by the presence of both biopolymers. Starting with the 25–75 CH–G system (Figure 6D,D′), it can be noted how this hydrogel presents an internal structure more similar to the structure of the unitary type A gelatin-based hydrogel. On the other hand, the structure of the 75–25 CH–G hydrogel (Figure 6B,B′) presents slight spherical grain sizes characteristic of the structure of the unitary chitosan-based hydrogel. Furthermore, the existing increase in pore diameter and the decrease in wall thickness with respect to type A gelatin unitary systems (Figure 6E,E′) can be highlighted.

Finally, the 50–50 CH–G hydrogel (50% chitosan and 50% type A gelatin) (Figure 6C,C′) has an intermediate internal structure between the two unitary hydrogels. The microstructural results of these hydrogels (in which chitosan and type A gelatin are involved in an intermediate proportion) could be compared with those obtained in another study completed by Mao et al. [45]. Specifically, the pore size shown in Figure 6 from the 50–50 CH–G hydrogel is smaller than that obtained by other techniques, such as the traditional manufacturing method of foaming used in the research of Mao et al. [45].

Therefore, it is possible to verify the importance of the processing method and working temperature in the case of pore interconnectivity. Thus, it allows for concluding how the systems will be more similar to the majority biopolymer.

### 2.4. Release Kinetic of the Tetracycline-Loaded Chitosan–Type-A-Gelatin Hydrogels

**Figure 7:**
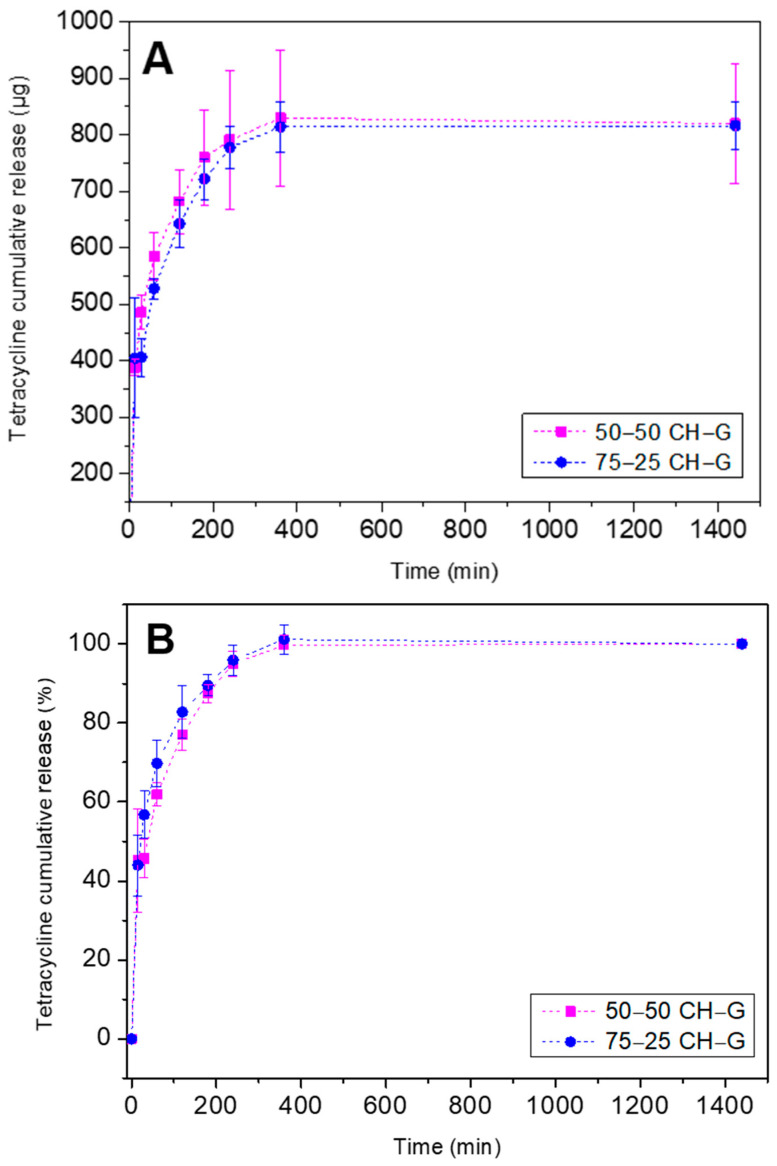
Evolution of the release kinetic of tetracycline-loaded hydrogels (50–50 CH–G and 75–25 CH–G) at 37 °C. (**A**) Amount of tetracycline released, (**B**) Percentage of tetracycline released.

Once the hydrogels were characterized, the 50–50 CH–G and 75–25 CH–G were selected as optimal due to their good properties, such as thermal stability and mechanical resistance, to evaluate the capability of chitosan–type-A-gelatin hydrogel to act as a drug delivery carrier. Specifically, the tetracycline release kinetics was evaluated over a period of 24 h and the results are shown in Figure 7A.

It can be noted that the release profile of both systems was very similar. Both hydrogels had a similar degradation mechanism due to the similar values obtained for parameter *n*. Specifically, the value of *n* in both cases was lower than 0.5 (Table 2), which indicates a typical Fickian diffusion law mechanism caused by the concentration gradient of tetracycline demonstrated by Korsmeyer et al. [45] and Lee [46]. In fact, diffusion-controlled mechanisms are the most commonly used to describe the drug release from the hydrogel. Fick’s law of diffusion, employing either constant or variable diffusion coefficients, is frequently used to model this type of release [47]. Particularly, the Fickian diffusion law explains the solute transport from polymer matrices, and it explains that the Fickian diffusion occurs when the polymer’s relaxation time is significantly longer than the characteristic solvent diffusion time. Moreover, it is important to highlight that the degradation of the hydrogel occurs in two steps. Initially, water immerses into the amorphous section of the hydrogel, causing random hydrolytic breaking of labile bonds. The second step begins once most of the amorphous regions have been degraded [48].

Regarding the total amount of tetracycline released, it can be seen that the hydrogels were able to cause a controlled release for 6 h, with an exponential profile being observed before reaching a stable zone. Thus, the total amount of tetracycline released during this time was similar for both systems, resulting in an average amount of tetracycline released of 800 µg which corresponds with the 100% of release shown in Figure 7B. This period of release could be of interest in the case of using hydrogels in biomedical applications, which requires an early and quick medication release [32]. For example, this kind of release could be essential to combat the possible inflammation and infections that occur just after the surgery, avoiding premature implant failure [49].

Finally, comparing the systems with double cross-linked (ionic and covalent) gelatin type A and chitosan hydrogels tested [50], loaded with caffeine, exponential release profiles can be observed, in which the amount of the model drug is around 300 mg/g of dry hydrogel at 24 h. Thus, it highlights the importance of the process carried out for the synthesis and its influence on the polymeric density of the network.

## 3. Conclusions

Hybrid hydrogels combining chitosan and type A gelatin in different proportions were successfully developed, demonstrating superior mechanical properties, thermal stability, and microstructure compared to unitary systems. Particularly, the hydrogels obtained with a small amount of chitosan demonstrated an enhanced thermal stability and better mechanical resistance which make them suitable materials for biomedical applications.

The study highlighted the potential synergistic effects of combining both biopolymers with 3 wt.%, as evidenced by the microstructural analysis and their rheological and chemical properties. The processing method effectively allowed the necessary bond formations, as shown by the FTIR spectra. Furthermore, rheological tests demonstrated an improvement in the mechanical properties compared to the unitary systems, such as an improvement in the thermal stability of type A gelatin hydrogels, allowing its application at temperatures of 40 °C without degradation.

Due to their good properties, such as thermal stability and mechanical resistance, 50–50 CH–G and 75–25 CH–G hydrogels were selected as the optimal hydrogels to evaluate their capability to act as drug carriers. In this case, the release obtained indicates a typical Fickian diffusion law mechanism and took only 6 h. Therefore, this release time is intended to be optimized to avoid a rapid and undesired release, allowing a longer release time to be achieved.

For this purpose, future studies should focus on optimizing the release time by incorporating different crosslinking agents and combining them with other biopolymers. Additionally, a textural study is necessary to quantify the smoothness and roughness of hydrogels, among other parameters. Moreover, it could be essential to modify the tetracycline integration to avoid possible light degradation throughout the process. Finally, the in vivo efficacy of these hydrogels should also be characterized to determine the low toxicity, anti-inflammatory, and antibacterial properties of the chitosan and the biocompatibility of the gelatine.

## 4. Materials and Methods

### 4.1. Materials

Low-molecular-weight chitosan (CH) (MW = 130,000 g/mol and deacetylation degree 75–85%) from Sigma–Aldrich S.A. (Taufkirchen, Germany) and Type A gelatin (G) (gel strength ca. 300) from Sigma–Aldrich S.A. (Darmstadt, Germany) were used as the raw materials for the development of hybrid hydrogels. Moreover, an acetic acid solution with a concentration of 0.05 M and a pH of 3.2 was used as a polymerization solvent. On the other hand, to increase the pH of the chitosan and achieve a total gelation of the hydrogels, a 6 M sodium hydroxide (NaOH) was also used. Acetic acid and NaOH were both obtained by Panreac Química S.A. (Barcelona, Spain). Tetracycline was also used in this work and supplied by Sigma–Aldrich S.A. (Darmstadt, Germany).

### 4.2. Synthesis of Chitosan–Gelatin Type A-Based Hydrogel

The synthesis of chitosan–type-A-gelatin hydrogels were carried out using a gelation process whose protocol is similar to the one previously defined by Perez-Puyana et al. [40]. The hydrogels were prepared at a concentration of 3 wt.% using different ratios of CH–G (100–0, 75–25, 50–50, 25–75 and 0–100) in 0.05 M acetic acid [41]. This detailed composition of the hydrogels is crucial for understanding the gelation process.

Briefly, to prepare a hydrogel, an appropriate amount of both biopolymers was initially weighted and dissolved in 40 mL of 0.05 M acetic acid in a 100 mL glass beaker, magnetically stirred at 300 rpm and 50 °C for 1 h. The compositions of the prepared hydrogels are given in Table 3.

**Figure 8:**
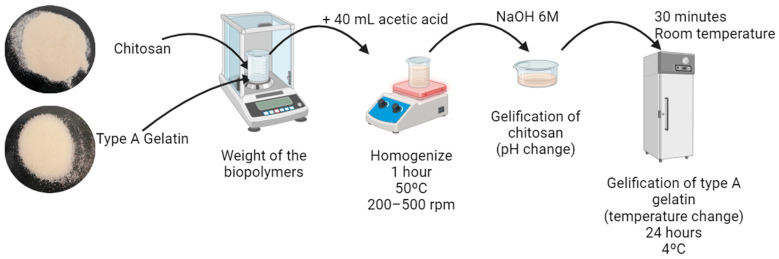
Synthesis of Chitosan–Gelatin Type A-based hydrogel.

Later, to achieve the gelation of both materials, it is necessary to change the pH that causes the gelling of chitosan and drop the temperature to help the gelation of type A gelatin. Particularly, in this work, first, the pH was changed from 3.2 to 7.0 by adding 6 M NaOH sprayed and then, the solution was placed in a fridge with the aim of cooling the sample for 24 h at a temperature of 4 °C. Finally, in Figure 8, a scheme of the process of synthesis of the hybrid hydrogels is represented.

### 4.3. Characterization of Hydrogels

#### 4.3.1. Chemical Characterization: Fourier Transform Infrared Spectroscopy (FTIR)

A Fourier Transform Infrared Spectroscopy (FTIR) analysis was conducted using a FTIR-4100 spectrophotometer manufactured by Jasco in Tokyo, Japan. The objective was to identify the different chemical bonds present in the systems. Analyzing the differences between the systems becomes possible by determining the bonds comprising each system, as each bond emits at different wavelengths. The spectra were obtained for a wavenumber spectrum ranging from 2000 to 750 cm^−1^ and were processed with Jasco Spectra ManagerTM software, version 2.

#### 4.3.2. Rheological Characterization

In order to examine the mechanical properties of the developed hydrogels, different rheological tests were performed by using a controlled-stress oscillatory rheometer AR2000 (TA Instruments, New Castle, DE, USA) equipped with a parallel serrated plate–plate geometry (diameter: 40 mm). Five different measurements were carried out:

- Strain sweep tests: Measurements among 0.1% and 100% strain at 1 Hz and temperatures of 5 °C and 40 °C were evaluated in order to determine the linear viscoelastic range. The purpose of these tests was to ensure that the strain values used were consistently below the critical strain, which is the maximum strain value that the sample can withstand within the linear viscoelastic range.
- Time sweep tests simulating gelation process: The gelation process of the hydrogels was studied by performing time sweep tests at 5 °C (1% strain and 1 Hz), simulating the conditions of the synthesis of hydrogels previously described.
- Frequency sweep tests: These tests were evaluated in a frequency range between 0.02 and 20 Hz, at a constant strain of 1% (within the linear viscoelastic range) and different temperatures (5 °C and 40 °C). The evolution of the elastic and viscous moduli (G′ and G″, respectively) was obtained and evaluated, as well as the loss tangent (tan δ = G″/G′). Moreover, the elastic modulus and the loss tangent were obtained and analyzed at a frequency of 1 Hz to improve the comparison between the systems (G′_1_ and tan (δ)_1_, respectively).
- Temperature ramp tests: Temperature ramps between 5 and 40 °C were carried out to study the influence that temperature has on the stability of the hydrogels. These tests were performed at a constant strain of 1% and a frequency of 1 Hz. It is important to note that the critical temperature is the one at which the elastic modulus decreases significantly.
- Time sweep tests: In order to study the resistance of the hydrogel at a specific temperature, time sweep tests were evaluated at 40 °C during a time interval of 5 min with a constant strain of 1% and at a frequency of 1 Hz.

#### 4.3.3. Microstructural Characterization: Scanning Electron Microscopy (SEM)

The microstructural characterization of the different hydrogels was evaluated using a Zeiss EVO scanning electron microscope (Jena, Germany) with a voltage of acceleration of 10 kV and a magnification of 30×. However, due to their high content of water, these systems need a previous treatment, which consists of fixing the system with glutaraldehyde and osmium, followed by chemical drying based on acetone solutions. Moreover, the systems were coated with a film of Au/Pd in a high-resolution sputter coater, Leica (Wetzlar, Germany), to enhance their conductivity and, consequently, their visibility in the microscope.

#### 4.3.4. Release Kinetic of the Tetracycline-Loaded Chitosan–Type-A-Gelatin Hydrogels

In order to assess the effectiveness of the hydrogels as a drug delivery system, a concentration of 2 mg/mL of tetracycline was incorporated into the mixture. Tetracycline was added along with chitosan and type A gelatin before being homogenized. In this case, the volume of acid acetic added was 10 mL, so the total amount of tetracycline was 20 mg, which indicates a concentration per hydrogel mass of 1.77 mg/g. Thus, the solution was then magnetically stirred at 300 rpm but at a lower temperature of 35 °C due to the thermal degradability of tetracycline. The synthesis temperature of the hydrogels was modified due to the existence of degradation of the compound with both light and temperature, and it was recommended that the commercial flask was not to exceed temperatures of 37 °C.

Afterward, the gelation process was carried out as previously described. Once the hydrogels were formed, the release of tetracycline was examined.

To evaluate the release of the tetracycline, the hydrogels were placed into a recipient with 5 mL of Phosphate Buffer Solution (PBS) and covered with aluminum foil to avoid the degradation of tetracycline due to its photosensitivity. Subsequently, the systems were placed into a shaker incubator with a stirring speed of 50 rpm and a temperature of 37 °C, simulating blood flow movement and body temperature, respectively, for 24 h. At different time intervals of 15, 30, 60, 120, 180, 240, 360, and 1440 min, 500 µL of the solution was removed, and the concentration of tetracycline was analyzed using a Multi-Mode Microplate Reader (BioTek, Winooski, VT, USA) at a wavelength of 363 nm with *n* ≥ 4 replicates. After obtaining each measurement, the medium was replaced with an equivalent amount of PBS.

Finally, the drug release mechanism was modeled following the equation defined by Korsmeyer et al. [45] for polymeric systems:

(1) $$L=k\cdot t^{n}$$

where *L* is the degradation of the system and *t* indicates the period of the time of release. However, from this equation two different parameters were obtained. On the one hand, *n* which indicates the degradation mechanism that was taking place, and on the other hand *k* which indicates its kinetics.

#### 4.3.5. Statistical Analysis

The measurements of this study were obtained by repeating the experiments twice. The statical parameters were shown as mean ± standard deviation. Particularly, statistical analyses were obtained using the Student’s *t*-test and a unidirectional analysis of variance (ANOVA, *p* < 0.05) using the SPSS 18 statistical package.

## Figures and Tables

**Table 1:** Critical strain, G′ and tan δ at 1 Hz (indicated as G′_1_ and tan (δ)_1_) and critical temperature obtained for CH–G hydrogels with different biopolymers’ proportions at two temperatures, 5 °C and 40 °C.

System CH–G	Temperature (°C)	Critical Strain (%)	G′_1_ (Pa)	tan (δ)_1_ (-)	Critical Temperature (°C)
0–100	5	25.0 ± 0.6	1160 ± 253	0.029 ± 0.007	32.5–35.0
	40	100.2 ± 0.1	0.40 ± 0.01	8.85 ± 1.49	
25–75	5	100.8 ± 0.1	877 ± 145	0.027 ± 0.004	31.0–36.0
	40	100.6 ± 0.1	182 ± 56	0.032 ± 0.006	
50–50	5	65.3 ± 0.5	3880 ± 528	0.021 ± 0.001	35.0–40.0
	40	64.0 ± 0.5	710 ± 26	0.032 ± 0.003	
75–25	5	66.1 ± 0.4	1646 ± 56	0.072 ± 0.001	>40.0
	40	41.3 ± 0.4	1574 ± 38	0.090 ± 0.014	
100–0	5	10.4 ± 0.2	3907 ± 1106	0.117 ± 0.006	>40.0
	40	6.8 ± 0.5	10,262 ± 5810	0.088 ± 0.008	

**Table 2:** Kinetic constant (*k*) and degradation mechanism (*n*) obtained for the hydrogels as well as the amount of tetracycline released.

System (CH-G)	*k* (min^−n^)	*n*
50–50	32.5 ± 5.9	0.17 ± 0.03
75–25	36.4 ± 5.8	0.16 ± 0.02

**Table 3:** Compositions of CH–G-based hydrogels.

System CH–G	Weight of CH (g)	Weight of G (g)	Volume of Acetic Acid (mL)
0–100	0	1.2	40
25–75	0.3	0.9	40
50–50	0.6	0.6	40
75–25	0.9	0.3	40
100–0	1.2	0	40

## Data Availability

All data and materials are available on request from the corresponding author. The data are not publicly available due to ongoing researches using a part of the data.
